# Zhenban Rootstock Optimizes Shatian Pomelo Fruit Quality by Coordinating Primary and Secondary Metabolism

**DOI:** 10.3390/metabo16090697

**Published:** 2026-09-21

**Authors:** Xueyu Cui, Yujiao Peng, Zhuoming Liang, Xiao Du, Wenwen Huang, Lin Lin, Xiaoyu Zhang, Bing Wang, Chen Liu, Xiliang Xu

**Affiliations:** 1 Key Laboratory of Environment Change and Resources Use in Beibu Gulf, Ministry of Education, Nanning Normal University, Nanning 530001, China; 20180702@nnnu.edu.cn (X.C.); 20180718@nnnu.edu.cn (Y.P.);; 2 Yulin Academy of Agricultural Sciences, Yulin 537000, China; 3 School of Food Engineering, Harbin University of Commerce, Harbin 150028, China

**Keywords:** *Citrus maxima*, grafting, fruit quality, ascorbic acid, multiomics

## Abstract

Background/Objectives: Rootstock application is a critical strategy for improving citrus fruit quality, yet conventional combinations frequently suffer from an inherent sugar and acid imbalance. This study aimed to comprehensively evaluate the physiological and molecular impacts of three diverse rootstocks, including Trifoliate orange (Pt), Sour pomelo (Cg), and Zhenban pomelo (Zb), on Shatian pomelo to identify optimal grafting combinations and elucidate the underlying mechanisms governing superior fruit quality. Methods: Shatian pomelo scions were grafted onto three distinct rootstocks (Pt, Cg, and Zb) and compared against self-rooted Shatian pomelo plants (CK) under identical field conditions. We conducted a comparative physiological evaluation of specific fruit traits, explicitly examining single fruit weight, total soluble solids, dry matter content, as well as the dynamic accumulation of individual sugars, citric acid, and ascorbic acid. To uncover the underlying molecular regulatory networks, we integrated comprehensive transcriptomic and metabolomic analyses of the developing fruits, mapping the precise gene expression profiles and metabolite fluxes across the different treatments. Results: While the Pt and Cg rootstocks induced nonspecific metabolic fluctuations, the Zb rootstock uniquely optimized internal fruit quality without compromising fundamental fruit morphology. Zb grafted fruits exhibited superior sweetness, significantly reduced citric acid, and massively elevated ascorbic acid content. Integrative multiomics data revealed that Zb orchestrates a highly precise resource reallocation strategy. By transcriptionally suppressing energy-consuming pathways, specifically glycolysis and broad flavonoid biosynthesis, the Zb rootstock efficiently conserved vital structural carbon skeletons. This redirected carbon flux was seamlessly coordinated with the robust upregulation of SWEET sugar transporters and key enzymes in the L galactose pathway, thereby maximizing carbohydrate sink strength and vitamin C production. Concurrently, the downregulation of specific degradation enzymes ensured the stability of these targeted nutritional pools. Conclusions: The targeted carbon reallocation driven by the Zb rootstock effectively overcomes the traditional flavor bottlenecks in pomelo cultivation. These mechanistic insights not only highlight a highly efficient agricultural strategy for citrus improvement but also advance our fundamental understanding of metabolic regulation in grafted plants.

## 1. Introduction

Shatian pomelo (*Citrus maxima* (Burm.) Merr. cv. Shatian Yu) is widely cultivated in southern China and is highly prized for its unique flavor, rich nutritional profile, and significant economic value. In modern consumer markets, fruit quality is the most critical determinant of commercial success. The sensory quality and nutritional value of pomelo fruits are primarily dictated by the delicate balance of soluble sugars and organic acids, as well as the abundance of bioactive health-promoting compounds, particularly ascorbic acid (Vitamin C) [1,2]. High sugar content, optimal low acidity, and elevated antioxidant capacity have become the primary targets for premium citrus production [3]. However, the complex genetic background and long juvenile period of pomelo limit the rapid improvement of these polygenic traits through traditional breeding. Consequently, optimizing pre-harvest agronomic practices, particularly the selection of compatible and superior rootstocks, has emerged as a highly efficient strategy to manipulate fruit quality and maximize agricultural profitability [4].

Grafting is an ancient and indispensable horticultural technique universally employed in commercial citrus orchards. Rootstocks not only provide physical support and confer resistance against soil-borne biotic and abiotic stresses but also profoundly regulate scion vigor, yield, and internal fruit quality [5]. The rootstock-scion interaction involves a complex systemic exchange of water, mineral nutrients, phytohormones, and macromolecules. Previous studies have demonstrated that different rootstocks can significantly alter carbon allocation, primary metabolism, and secondary metabolite accumulation in the grafted scion [4,5]. While conventional rootstocks, such as *Poncirus trifoliata*, are widely used due to their broad adaptability and disease resistance, they often fall short in maximizing the organoleptic and nutritional potential of elite scion cultivars. In recent years, indigenous or locally selected rootstocks (such as Zhenban pomelo) have garnered increasing attention. These regional rootstocks, adapted through long-term natural and artificial selection, frequently exhibit exceptional compatibility with local elite cultivars, driving superior physiological performance. Nevertheless, the precise metabolic shifts and molecular regulatory networks governing how these specific local rootstocks optimize fruit quality remain largely elusive.

The intrinsic flavor and nutritional profile of citrus fruits are fundamentally determined by the orchestrated regulation of complex metabolic pathways within the fruit sink [3]. During fruit maturation, photoassimilates synthesized in source leaves are translocated primarily in the form of sucrose to the fruit, where they are unloaded and partitioned into hexose pools (glucose and fructose) via the coordinated action of sucrose transporters (SUTs), SWEET proteins, and invertases [6,7]. Simultaneously, fruit acidity in pomelo is predominantly determined by the accumulation and subsequent degradation of citric acid within the vacuole, a process tightly governed by the tricarboxylic acid (TCA) cycle and key enzymes such as citrate synthase and aconitase [8,9]. Furthermore, ascorbic acid, a critical non-enzymatic antioxidant and nutritional index, is synthesized through intricate multi-branched networks, primarily the L-galactose pathway and the D-galacturonate pathway [10]. Understanding how rootstock-derived signals coordinate the interplay among carbohydrate influx, organic acid catabolism, and ascorbate biosynthesis is crucial for deciphering the physiological mechanisms of quality enhancement.

Historically, the evaluation of rootstock effects has heavily relied on macroscopic phenotypic observations and targeted physiological measurements, which provide a fragmented view of the underlying biology. The advent of high-throughput multi-omics technologies has revolutionized the study of complex horticultural traits. Integrated transcriptomic and metabolomic analyses offer a robust systems-biology framework to bridge the gap between genotype and phenotype [11]. By coupling global gene expression profiling with the comprehensive quantification of primary intermediates (e.g., fructose-2-phosphate, TCA cycle organic acids) and secondary metabolites (e.g., flavonoids, glycosides), researchers can accurately map the metabolic fluxes and identify key regulatory hubs responsible for specific quality traits. This multi-level approach allows for a panoramic understanding of the rootstock-mediated transcriptional reprogramming and subsequent metabolic rewiring in scion fruits [11].

In the present study, we hypothesized that the local rootstock (Zhenban pomelo, Zb) induces a systemic physiological optimization in Shatian pomelo, leading to the targeted redirection of metabolic fluxes towards sugar accumulation, acid degradation, and antioxidant biosynthesis, thereby outperforming conventional rootstocks such as Trifoliate orange and Sour pomelo. To test this hypothesis, a comprehensive comparative evaluation was conducted using Shatian pomelo grafted onto different rootstock genotypes. We systematically determined the macroscopic fruit quality traits and primary nutritional indices. More importantly, we integrated transcriptomic sequencing with widely targeted metabolomics to delineate the internal regulatory landscape. By performing correlation network analyses between key differentially expressed genes and differential accumulated metabolites, we aimed to construct a precise biological working model. The objective of this research is to systematically evaluate the impact of these distinct rootstocks on fruit quality and to elucidate the underlying molecular and metabolic mechanisms driving phenotypic variations.

## 2. Materials and Methods

### 2.1. Plant Materials and Sampling

The Shatian pomelo scions were grafted onto three different rootstocks: *Poncirus trifoliata* (Pt), *Citrus grandis* (Cg), and a superior local rootstock, Zhenban pomelo (Zb). Self-rooted Shatian pomelo plants were used as the control (CK). All experimental trees were approximately ten years old and were cultivated following uniform standard orchard management protocols, ensuring consistent fertilization, irrigation, and pest control regimens across all treatment groups. Fruits of Shatian pomelo (*Citrus maxima* (Burm.) Merr. cv. Shatian Yu) were collected at the commercial maturation stage in November 2025 from an experimental orchard located in Rong County, Yulin City, Guangxi Zhuang Autonomous Region, China. The commercial maturity stage of Shatian pomelo was strictly defined in accordance with the local agricultural industry standards of the primary production regions and traditional harvesting practices. Specifically, the fruits were harvested in early November, coinciding with the traditional Frosts Descent period, when the total soluble solids (TSS) content reached the established commercial threshold of 11 to 12%. From each tree, ten representative fruits free of mechanical damage and disease symptoms were harvested. For each treatment group, three healthy and uniform trees were selected as biological replicates. The pulp tissues were immediately separated, frozen in liquid nitrogen, and stored at −80 °C for subsequent physiological assays and multi-omics analyses.

### 2.2. Determination of Total Titratable Acidity and Total Reducing Sugars

The total titratable acidity (TA) was determined using the classical acid-base titration method. Briefly, 10.0 g of homogenized pomelo pulp was diluted with distilled water to a final volume of 50 mL and filtered. A 10 mL aliquot of the filtrate was titrated with a standardized 0.1 M NaOH solution using 1% phenolphthalein as an indicator until a persistent faint pink color appeared for 30 s. The total acid content was calculated and expressed as mg/g fresh weight (FW).

The total reducing sugar content was quantified using the 3,5-dinitrosalicylic acid (DNS) colorimetric assay. Approximately 1.0 g of frozen pulp was extracted in 10 mL of distilled water at s100 °C for 30 min. After centrifugation, 1 mL of the supernatant was mixed with 2 mL of DNS reagent, boiled for 5 min, and rapidly cooled in an ice bath. The absorbance was measured at 540 nm using a UV-Vis spectrophotometer (Shanghai Jinghua Technology Instrument Co., Ltd., Shanghai, China). A standard curve was constructed using authentic D-glucose, and the results were expressed as mg/g FW.

### 2.3. Extraction and Quantification of Soluble Sugars, Citric Acid and Ascorbic Acid

The individual soluble sugar components (sucrose, glucose, and fructose) and citric acid were extracted and quantified using High-Performance Liquid Chromatography (HPLC) equipped with an Agilent 1260 Infinity II system (Agilent Technologies, Santa Clara, CA, USA). Briefly, 1.0 g of finely ground frozen pulp was extracted with 10 mL of 80% (*v*/*v*) aqueous ethanol in a water bath at 80 °C for 30 min. The homogenate was centrifuged at 10,000× *g* for 15 min at 4 °C. The extraction was repeated twice, and the supernatants were pooled, evaporated to dryness under a vacuum, and re-dissolved in 2 mL of ultrapure water. The final solution was filtered through a 0.22 μm nylon membrane filter prior to injection.

For soluble sugar analysis, separation was performed on an Agilent ZORBAX Carbohydrate (NH2) analytical column (4.6 × 250 mm, 5 μm). The mobile phase consisted of acetonitrile and ultrapure water (75:25, *v*/*v*) with an isocratic elution at a flow rate of 1.0 mL/min. The column temperature was maintained at 35 °C, and detection was achieved using a Refractive Index Detector (RID).

For citric acid quantification, the separation was conducted on an Agilent ZORBAX Eclipse Plus C18 column (4.6 × 250 mm, 5 μm). The mobile phase was 0.1% (*v*/*v*) phosphoric acid solution running isocratically at a flow rate of 1.0 mL/min. The column temperature was set at 25 °C, and the eluent was monitored by a Diode Array Detector (DAD) at a wavelength of 210 nm. Compounds were identified and quantified by comparing their retention times and peak areas with those of high-purity commercial standards. The content of citric acid was expressed as nmol/g FW, and the specific sugars were expressed as mg/g FW.

To prevent oxidation during extraction, the ascorbic acid (Vitamin C) content was assessed using an optimized HPLC method. Approximately 2.0 g of frozen sample was homogenized with 10 mL of ice-cold 2% (*w*/*v*) metaphosphoric acid solution. The mixture was extracted in the dark at 4 °C for 15 min and then centrifuged at 12,000× *g* for 15 min at 4 °C. The resulting supernatant was filtered through a 0.22 μm aqueous syringe filter. The HPLC analysis was conducted using a reverse-phase C18 column (4.6 × 250 mm, 5 μm) with an isocratic elution of 0.1% (*v*/*v*) oxalic acid in water. The flow rate was 1.0 mL/min, and the column temperature was 25 °C. The detection wavelength for the DAD was set at 245 nm. The ascorbic acid concentration was determined based on a highly linear calibration curve of the standard (R^2^ > 0.999) and expressed as μg/g FW.

### 2.4. Transcriptomic Analysis

#### 2.4.1. RNA Extraction and Library Preparation

Total RNA was extracted from the fruit pulp samples. To ensure the reliability of downstream library construction, RNA concentration and integrity were precisely evaluated utilizing a Qubit 4.0 Fluorometer (Thermo Fisher Scientific, Waltham, MA, USA) and a Qsep400 Bioanalyzer (BiOptic Inc., New Taipei City, Taiwan, China), respectively. For eukaryotic library preparation, mRNA was enriched using oligo(dT) magnetic beads targeting the poly(A) tail. The enriched mRNA was subsequently sheared into short fragments using a fragmentation buffer. First-strand cDNA was synthesized using random hexamer primers, followed by second-strand cDNA synthesis utilizing DNA polymerase I, dNTPs, and buffer. After purification with magnetic beads, the double-stranded cDNA underwent end repair, A-tailing, and sequencing adapter ligation. Size selection of the adapter-ligated fragments was performed using magnetic beads, followed by PCR amplification to construct the final cDNA library. The library quality and insert size were assessed using a Qubit fluorometer and a Fragment Analyzer before sequencing.

#### 2.4.2. Sequencing and Data Processing

The qualified cDNA libraries were pooled according to the target data output and sequenced on the Illumina platform. To ensure high accuracy for downstream bioinformatics analyses, the raw reads were subjected to rigorous quality control using the fastp software (v0.23.2) [12]. The filtering criteria included the removal of adapter-containing reads, paired reads with an ambiguous nucleotide (N) content exceeding 10%, and paired reads in which more than 50% of the bases possessed a quality score of Q ≤ 20.

#### 2.4.3. Read Alignment and Quantification of Gene Expression

The resulting high-quality clean reads were mapped to the Shatian pomelo reference genome utilizing HISAT2 (2.2.1) [13]. Based on the alignment results and genomic positional information, the read counts for each gene were quantified using featureCounts [14]. To accurately reflect true transcript expression levels and eliminate the influence of sequencing depth and gene length, the expression abundance was normalized and calculated as Fragments Per Kilobase of transcript per Million mapped reads (FPKM).

#### 2.4.4. Differential Expression Analysis

Differential gene expression analysis between different rootstock treatment groups was performed using the DESeq2 R package, which is designed for experimental designs with biological replicates [15]. The resulting *p* values were adjusted using the Benjamini–Hochberg approach to control the False Discovery Rate (FDR). Genes with a specific adjusted *p* value and fold change threshold were defined as differentially expressed genes (DEGs).

### 2.5. Metabolomics Analysis

#### 2.5.1. Metabolite Extraction

The pulp samples were vacuum freeze-dried using a lyophilizer (Scientz-100F, Scientz Biotechnology Co., Ltd., Ningbo, China) for 63 h and subsequently crushed into a fine powder using a mixer mill (MM 400, Retsch GmbH, Haan, Germany) at 30 Hz for 1.5 min. Approximately 30 mg of the lyophilized powder was extracted with 1.5 mL of pre-cooled 70% aqueous methanol (containing internal standards) at −20 °C. To ensure thorough extraction, the mixture was vortexed for 30 s every 30 min for a total of six times. Following centrifugation at 12,000 rpm for 3 min, the supernatant was carefully collected, filtered through a 0.22 μm microporous membrane, and transferred to sample vials for subsequent UPLC-MS/MS analysis.

#### 2.5.2. UPLC-MS/MS Conditions

Metabolomic profiling was performed utilizing a UPLC-ESI-MS/MS system (ExionLC™ AD, SCIEX, Framingham, MA, USA), primarily composed of an ExionLC™ AD system coupled with a tandem mass spectrometer. Chromatographic separation was achieved on an Agilent SB-C18 analytical column (1.8 µm, 2.1 × 100 mm) maintained at 40 °C. The mobile phase consisted of 0.1% formic acid in ultrapure water (solvent A) and 0.1% formic acid in acetonitrile (solvent B). The gradient elution program was set as follows: 0–9.0 min, 5% to 95% B; 9.0–10.0 min, maintained at 95% B; 10.0–11.1 min, decreased to 5% B; and 11.1–14.0 min, equilibrated at 5% B. The flow rate was constant at 0.35 mL/min, and the injection volume was 2 μL.

For mass spectrometry, the electrospray ionization (ESI) source temperature was set at 500 °C. The ion spray voltage (IS) was configured at 5500 V for positive ion mode and −4500 V for negative ion mode. The ion source gas I (GSI), gas II (GSII), and curtain gas (CUR) were set at 50, 60, and 25 psi, respectively. The collision-induced dissociation (CID) parameter was set to high. The triple quadrupole (QQQ) mass spectrometer was operated in multiple reaction monitoring (MRM) mode with collision gas (nitrogen) set to medium [16]. The declustering potential (DP) and collision energy (CE) were optimized for individual MRM transitions.

#### 2.5.3. Metabolite Identification and Quantification

The qualitative analysis of primary and secondary metabolites was conducted based on the Metware Database (MWDB) according to their secondary spectral information. Isotopic signals, redundant adduct ions (e.g., K^+^, Na^+^, NH^4+^), and repetitive fragment ions derived from larger molecules were systematically excluded to ensure precise identification [17]. Metabolite quantification was accomplished using the MRM mode of the QQQ mass spectrometer, which filters precursor ions and selects specific product ions to eliminate non-target interference. Mass spectrometry data were processed using Analyst 1.6.3 software (Sciex, ExionLC™ AD, SCIEX, Framingham, MA, USA). The chromatographic peak area integration and correction for identical metabolites across different samples were performed using MultiQuant software (v3.0.3, SCIEX, Framingham, MA, USA) to determine the relative abundance of the metabolites [18].

#### 2.5.4. Differential Metabolite Analysis

To identify differentially accumulated metabolites across the various rootstock treatments, pairwise differential analyses were systematically conducted. Principal component analysis (PCA) and orthogonal partial least squares discriminant analysis (OPLS DA) were performed to evaluate the overall metabolic variations and extract the variable importance in projection (VIP) scores for individual metabolites. Differentially accumulated metabolites were rigorously defined using the following selection criteria: a VIP score ≥ 1.0 combined with an absolute Log_2_ fold change ≥ 1.0.

### 2.6. Statistical Analysis and Data Visualization

All quantitative data in this study were obtained from at least three independent biological replicates and are presented as the mean value standard deviation of the mean. Statistical analyses were performed using SPSS software (version 26.0, IBM Corp., Armonk, NY, USA). One way analysis of variance (ANOVA) followed by Duncan multiple range test was applied to determine significant differences among distinct grafting treatments, with a threshold of *p* < 0.05 considered statistically significant. For multiomics dataset processing, principal component analysis (PCA) and Venn diagrams were generated using R software (version 4.2.0) with the ggplot2 and VennDiagram packages. KEGG pathway classification and enrichment analyses were conducted using the clusterProfiler R package. Additional data visualizations, including heatmaps, bar graphs, and boxplots, were generated using the Seaborn (v0.11.2) and Matplotlib (v3.4.3) packages in Python (version 3.10).

## 3. Results

### 3.1. Physiological Optimization of Fruit Quality by Superior Rootstocks

**Figure 1:**
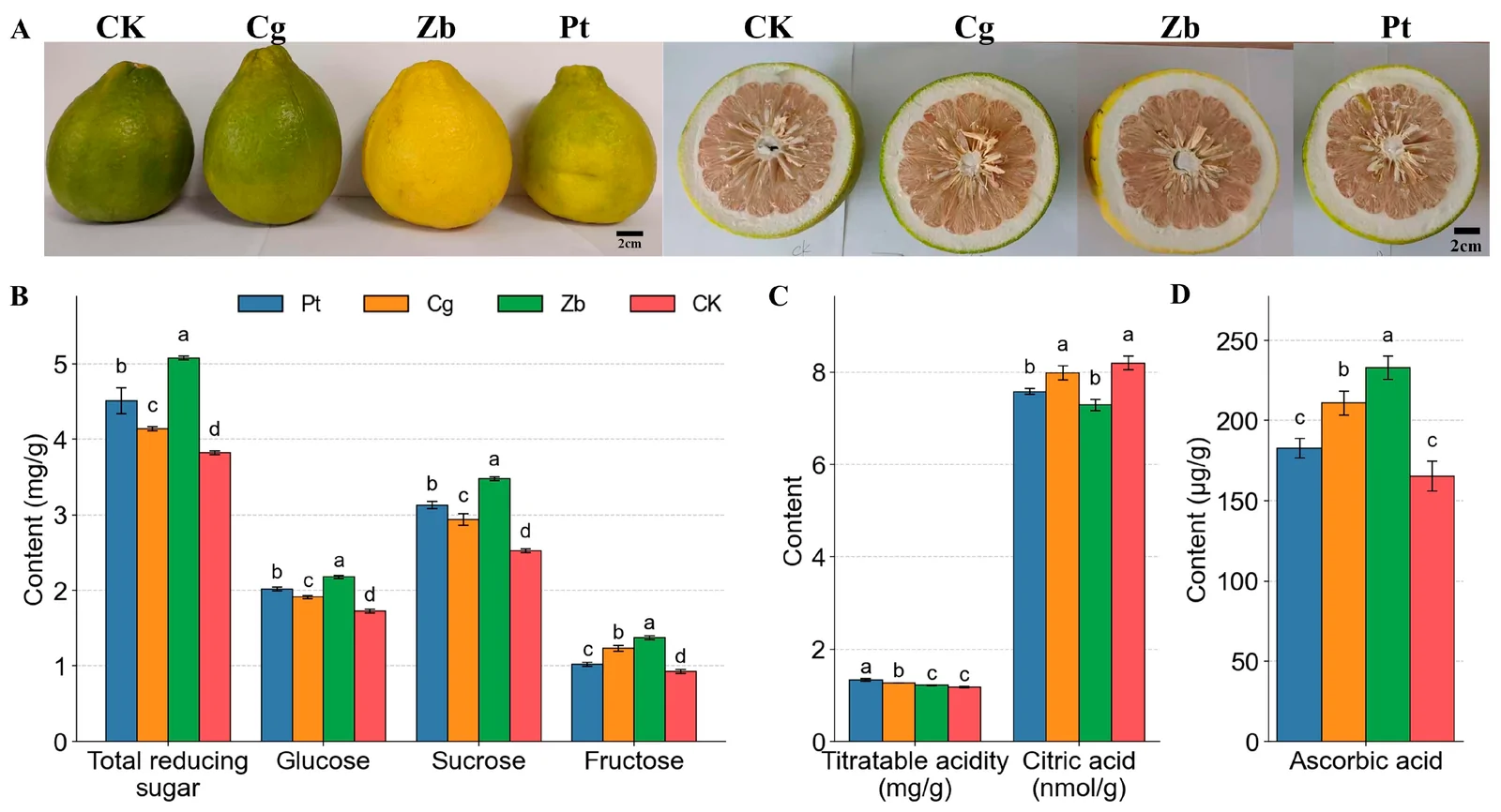
Rootstock-mediated effects on the morphological phenotypes and physiological quality of Shatian pomelo fruits. (**A**) Representative visual phenotypes of intact fruits (**left**) and equatorial cross-sections (**right**). (**B**) Quantitative profiling of soluble sugar accumulation. (**C**) Variations in organic acid pools. (**D**) Variations in the accumulation of the characteristic antioxidant metabolite, ascorbic acid. Treatments are defined as follows: CK, self-rooted Shatian pomelo; Cg, Sour pomelo rootstock; Zb, Zhenban pomelo rootstock; Pt, Trifoliate orange rootstock. Data are presented as the mean ± SD of three biological replicates. Different lowercase letters indicate significant differences at *p* < 0.05 according to Duncan multiple range test.

To evaluate the macroscopic effects of different local rootstocks on Shatian pomelo, the fundamental physiological indices of various graft combinations (Pt, Cg, Zb) and the control (CK) were first determined. The results showed that across all treatment groups, the single fruit weight ranged from 1.03 to 1.17 kg (Figure 1A and Table 1), and the total soluble solids remained stable between 12.4% and 12.5%. These metrics exhibited no significant differences compared to the CK group, indicating that the selected grafting rootstocks did not significantly alter the basic macroscopic morphology and total soluble solids of the pomelo fruits. However, the dry matter content of the treatment groups varied from 27.7% to 30.9% (Table 1), which was generally lower than the 34.6% observed in the CK group.

Following the assessment of the macroscopic physical traits, we further investigated the internal biochemical properties by profiling the accumulation of various soluble sugars. Biochemical quantitative analysis revealed that the total reducing sugar content in the Pt and Zb rootstock groups was significantly elevated to 4.51 mg/g and 5.08 mg/g, respectively, which were considerably higher than that of the CK (Figure 1B). Further dissection of the monosaccharide components showed that glucose enrichment was most pronounced in the Zb and Pt groups, with concentrations of 2.07 mg/g and 2.02 mg/g, respectively. Drastic fructose accumulation was mainly observed in the Zb and Cg groups, which reached levels of 1.29 mg/g and 1.23 mg/g, respectively. Meanwhile, the sucrose content varied distinctively between the treatments. Compared to the CK group, which exhibited a sucrose concentration of 2.5 mg/g, all rootstock treatments enhanced sucrose accumulation. Notably, the Zb group achieved the highest sucrose level at 3.5 mg/g, followed sequentially by the Pt and Cg groups. Notably, the Zb rootstock demonstrated the most outstanding physiological performance in enhancing glucose, fructose, and total reducing sugars, suggesting that this superior local rootstock intensely activated the sugar transport and unloading networks of the fruit to provide an abundant carbon skeleton for fruit quality formation.

In addition to sugar accumulation, the rootstocks profoundly altered the metabolic flow of organic acids, another key factor determining the intrinsic quality of citrus fruits. The titratable acidity content exhibited significant variations between the treatments. The Pt group showed the highest titratable acidity level, followed by the Cg group, whereas the Zb and CK groups maintained lower levels (Figure 1C). Consequently, the TSS/TA ratio, a critical determinant of comprehensive fruit flavor, exhibited corresponding variations. The TSS/TA ratio in the Zb rootstock group showed no significant difference from that of the self-rooted CK control, both maintaining a superior level. Conversely, the conventional Pt and Cg rootstocks induced a significant decline in this ratio. More importantly, citric acid, the core intermediate product of the tricarboxylic acid cycle, exhibited a strict descending gradient. The citric acid content reached its highest level at 8.20 nmol/g in the CK group, followed sequentially by 7.98 nmol/g in the Cg group and 7.58 nmol/g in the Pt group, and ultimately dropped to its lowest level of 7.28 nmol/g in the Zb rootstock group. Concurrently, accompanied by this decline in primary citric acid content, the content of ascorbic acid, a characteristic metabolite reflecting high fruit quality and stress resistance, experienced an explosive increase (Figure 1D). The average ascorbic acid content in the CK group was a mere 165.35 μg/g. In contrast, all rootstock treatments promoted its accumulation. The concentration increased notably in the Pt and Cg groups, with the latter reaching 210.96 μg/g, and surged to a peak of 232.86 μg/g in the Zb group.

### 3.2. Rootstock-Mediated Modulation of Global Metabolome and Specific Secondary Pathways

**Figure 2:**
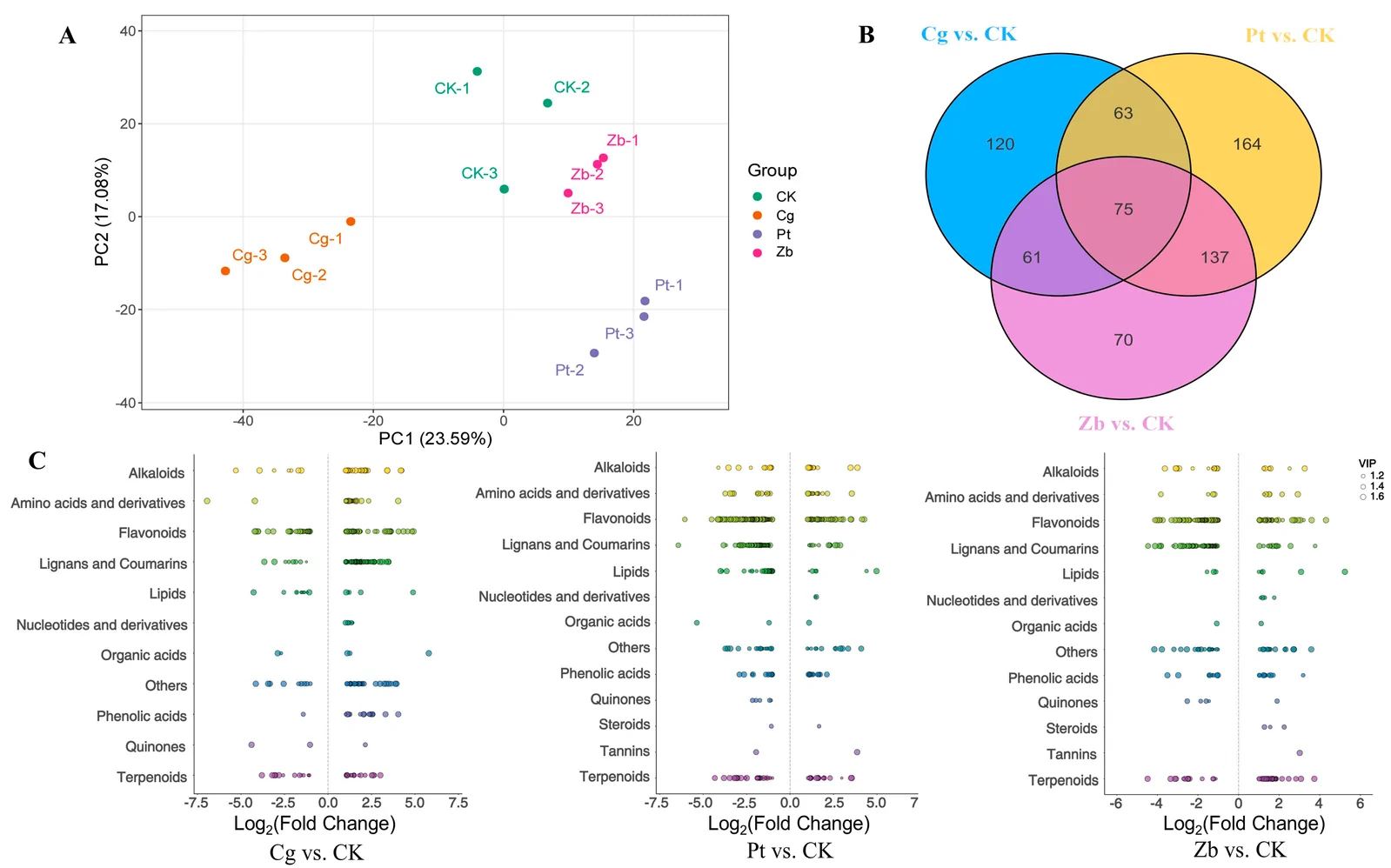
Global metabolomic profiling and distribution patterns of differentially accumulated metabolites across different rootstock treatments. (**A**) Principal component analysis plot. (**B**) Venn diagram. (**C**) Classification and abundance distribution of the differentially accumulated metabolites. The horizontal axis indicates the base-2 logarithm of the fold change for individual metabolites, which are categorized by distinct colors based on their primary biochemical classes. The size of each dot is strictly proportional to its Variable Importance in Projection (VIP) value. Treatments are defined as follows: CK, self-rooted Shatian pomelo; Cg, Sour pomelo rootstock; Zb, Zhenban pomelo rootstock; Pt, Trifoliate orange rootstock.

To systematically elucidate the systemic physiological and molecular basis underlying these rootstock-mediated optimizations, particularly the massive sugar accumulation and the dramatic ascorbic acid surge, global metabolomic profiling was performed on the Shatian pomelo fruits. Principal component analysis (PCA) was first conducted to assess the overall variance and distribution among the samples. As shown in the PCA score plot (Figure 2A), the three biological replicates of each group clustered tightly together, indicating high reproducibility and robust reliability of the dataset. The first two principal components, PC1 and PC2, accounted for 23.6% and 17.1% of the total variance, respectively. Notably, the three rootstock treatment groups were distinctly separated from the CK group along the principal components. This clear spatial segregation indicated that the introduction of different local rootstocks induced significant alterations in the overall metabolic profiles of the developing fruits.

To further quantify these molecular variations and identify the specific responsive factors, pairwise differential analyses were performed. A Venn diagram was constructed to illustrate the distribution of the overlapping and rootstock-specific differentially accumulated metabolites (DAMs) (Figure 2B). The intersection of the comparison groups revealed a core set of 75 common differential features shared by all rootstock treatments. Beyond this conserved core, the number of uniquely altered features varied distinctly among the specific rootstocks. The Pt group exhibited the highest number of unique features at 164, followed by 120 unique features in the Cg group. Conversely, the Zb rootstock group presented the lowest number, containing exactly 70 unique differential features.

To comparatively evaluate the impact of different rootstock genotypes on the global metabolic networks, the DAMs from the three grafted groups were plotted against the CK (Figure 2C). The Log_2_FC class scatter plots revealed that rootstock grafting universally elicited an extensive, class-specific reprogramming of both primary and secondary metabolism in Shatian pomelo fruits; however, the magnitude and direction of these shifts were highly dependent on the rootstock genotype. Classes such as flavonoids, lignans and coumarins exhibited a striking density of bidirectional DAMs. However, in Zb treatment, the highly specific up-regulation of certain metabolites was accompanied by a markedly more pronounced down-regulation of flavonoids, reflecting a strategic restriction of this energy-intensive pathway. Interestingly, while the majority of metabolic categories displayed a bidirectional regulation, nucleotides and derivatives emerged as a unique exception, being exclusively up-regulated across all three grafting treatments. However, the true distinctiveness of the Zb rootstock lies in its specific secondary metabolic signature. Crucially, steroids and tannins were strictly and exclusively up-regulated only in the Zb grafted fruits, a pattern completely absent in the Pt and Cg groups. This specific accumulation highlighted the unique capacity of the local rootstock to selectively activate specific functional pathways.

### 3.3. Rootstock-Mediated Diversion of Core Metabolites and Optimization of Fruit Quality

**Figure 3:**
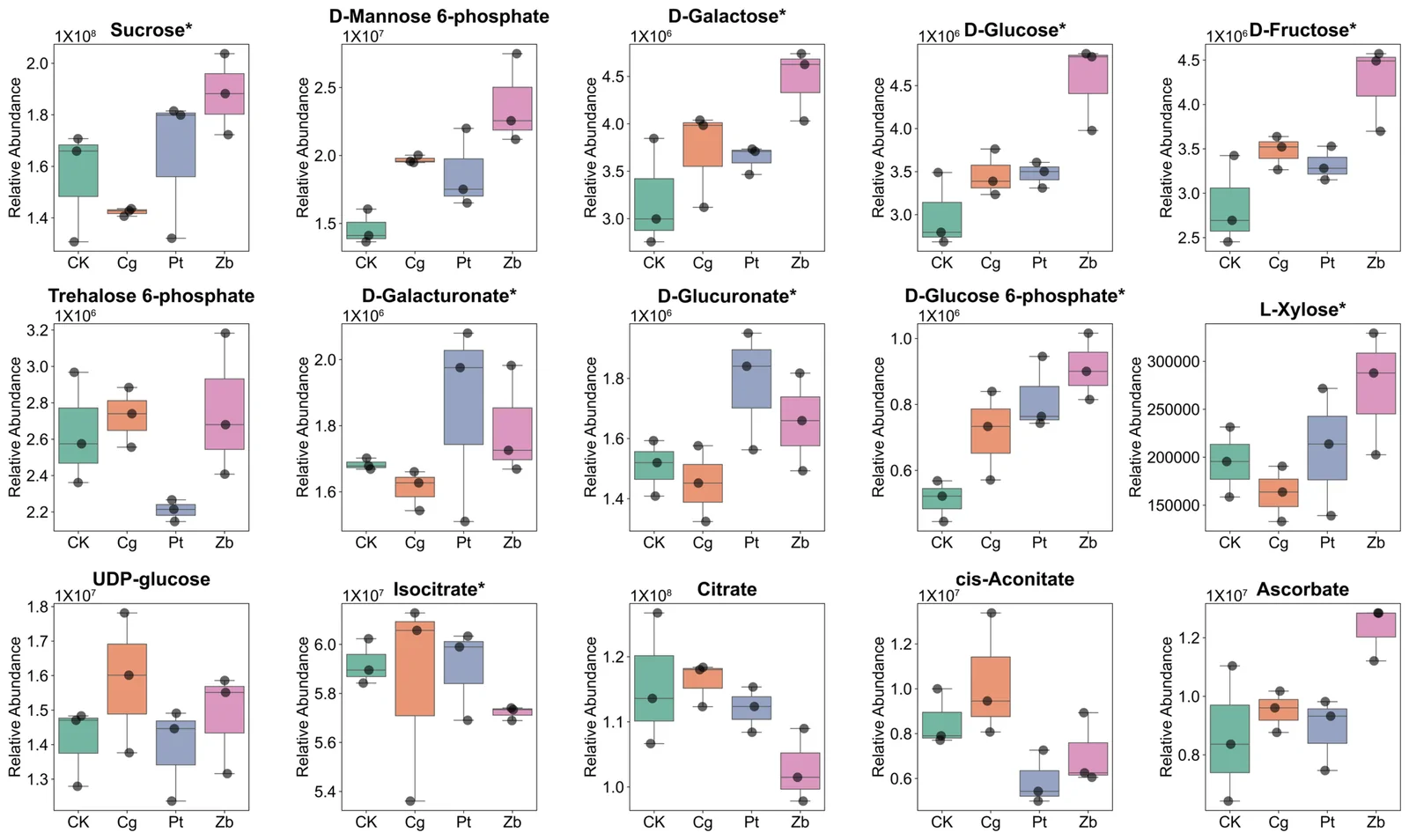
Relative abundances of key metabolites involved in carbohydrate metabolism and the TCA cycle. Boxplots illustrate the differential accumulation of representative sugars, sugar phosphates, organic acids, and ascorbate across the self-rooted control (CK) and three grafting treatments (Cg, Pt, and Zb). In each boxplot, the horizontal line denotes the median, while the upper and lower hinges correspond to the 75th and 25th percentiles, respectively. Whiskers extend to the maximum and minimum data points, and individual biological replicates are overlaid as black dots. Treatments are defined as follows: CK, self-rooted Shatian pomelo; Cg, Sour pomelo rootstock; Zb, Zhenban pomelo rootstock; Pt, Trifoliate orange rootstock. * indicates the presence of isomers.

To precisely decipher the specific impacts of different local rootstocks on the quality establishment of Shatian pomelo at the metabolic level, quantitative comparisons were focused on core intermediate metabolites (Figure 3). Regarding carbohydrate metabolism, soluble sugars and their phosphorylated intermediates underwent drastic positive accumulation in the rootstock-grafted groups. The Zb rootstock induced the highest accumulation of free mono- and disaccharides, notably surpassing the CK by 1.52-fold and 1.49-fold in the relative abundances of D-glucose and D-fructose, respectively (Figure 3). Concurrently, key phosphorylated intermediates in the glycolysis and hexose metabolism pathways also reached their peak levels in the Zb group, including D-glucose 6-phosphate, which surged by nearly 78% relative to the CK. Furthermore, various sugar derivatives such as D-galactose and L-xylose were highly elevated under the Zb treatment, whereas D-galacturonate recorded its maximum abundance in the Pt group.

In the tricarboxylic acid cycle pathway, the core organic acid nodes exhibited distinct abundance patterns. The relative abundances of citrate, cis-aconitate, and isocitrate all reached their highest levels in the Cg group. In sharp contrast, the Zb rootstock effectively downregulated this organic acid pool. Specifically, the citrate abundance dropped by over 11% in the Zb group compared to the control baseline, while cis-aconitate exhibited a pronounced 17% reduction relative to the control levels. The Pt rootstock similarly suppressed organic acid accumulation, notably recording the absolute lowest level of cis-aconitate among all groups (Figure 3).

Within the secondary antioxidant network, the abundance of ascorbate exhibited a clear and strict ascending gradient across the treatments. Metabolite quantification revealed that the control group maintained the lowest baseline abundance of ascorbate. Conversely, all rootstock treatments actively promoted its accumulation. The ascorbate abundance increased sequentially from the Pt group to the Cg group, and ultimately surged to a maximum level in the best-performing Zb group, achieving an impressive 43% increase over the control baseline (Figure 3).

### 3.4. Transcriptional Activation of Metabolic Networks by Superior Rootstock

**Figure 4:**
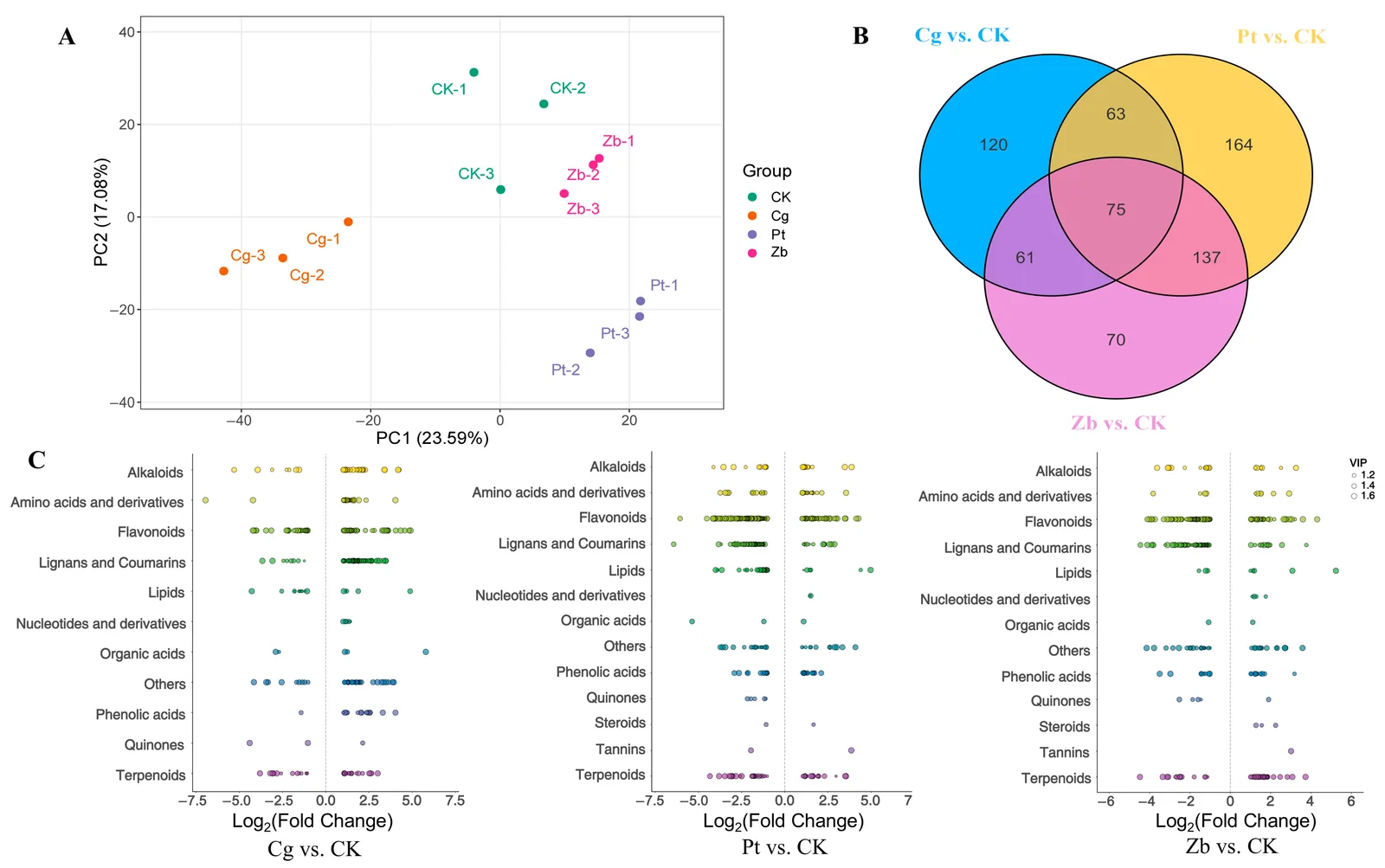
Identification and KEGG pathway classification of differentially expressed genes (DEGs) across different grafting treatments. (**A**) Principal component analysis plot. (**B**) Venn diagram. (**C**) Bar charts illustrating the KEGG pathway classification of DEGs in the Cg vs. CK, Pt vs. CK, and Zb vs. CK groups. The *y*-axis represents the specific KEGG pathways, and the *x*-axis represents the percentage and absolute number of DEGs assigned to each pathway. Treatments are defined as follows: CK, self-rooted Shatian pomelo; Cg, Sour pomelo rootstock; Zb, Zhenban pomelo rootstock; Pt, Trifoliate orange rootstock.

To elucidate the underlying genetic basis of the rootstock-mediated physiological and metabolic shifts, comparative transcriptomic analysis was performed on the fruit tissues. As illustrated in the Venn diagram (Figure 4A), grafting induced distinct and genotype-dependent transcriptional responses. Transcriptomic profiling revealed remarkably divergent expression patterns among the treatments. Notably, the Zb rootstock elicited the most extensive transcriptional modulation, generating a total of 616 differentially expressed genes (DEGs) compared to the self-rooted CK group. In contrast, the Pt and Cg rootstocks induced 422 and 50 DEGs, respectively. Crucially, 439 DEGs were strictly unique to the Zb treatment, whereas a mere 9 DEGs were universally shared across all three grafting combinations.

To decipher the biological functions of these rootstock-responsive genes, Kyoto Encyclopedia of Genes and Genomes (KEGG) pathway enrichment analyses were mapped for the respective comparison groups (Figure 4B,C). The functional mapping revealed that the regulatory dominance of the Zb rootstock was primarily concentrated in metabolic processes. Specifically, among the respective comparisons against the self-rooted CK, the Zb group exhibited the highest number of DEGs assigned to “Metabolic pathways” and “Biosynthesis of secondary metabolites” relative to both the Pt and Cg treatments. Furthermore, within these broad networks, the specific pathway of “Starch and sucrose metabolism” was notably enriched in the Zb-responsive gene set. This targeted transcriptional mobilization provided direct genetic evidence for the high sugar content observed at the physiological level. The unparalleled activation of these metabolic gene cascades driven by the Zb rootstock ultimately served as the molecular engine for the targeted reallocation of resources, facilitating the optimal accumulation of sugars and specific health-promoting compounds in the fruits.

### 3.5. Coordinated Transcriptional Reprogramming of Carbohydrate Mobilization and Ascorbate Metabolism

**Figure 5:**
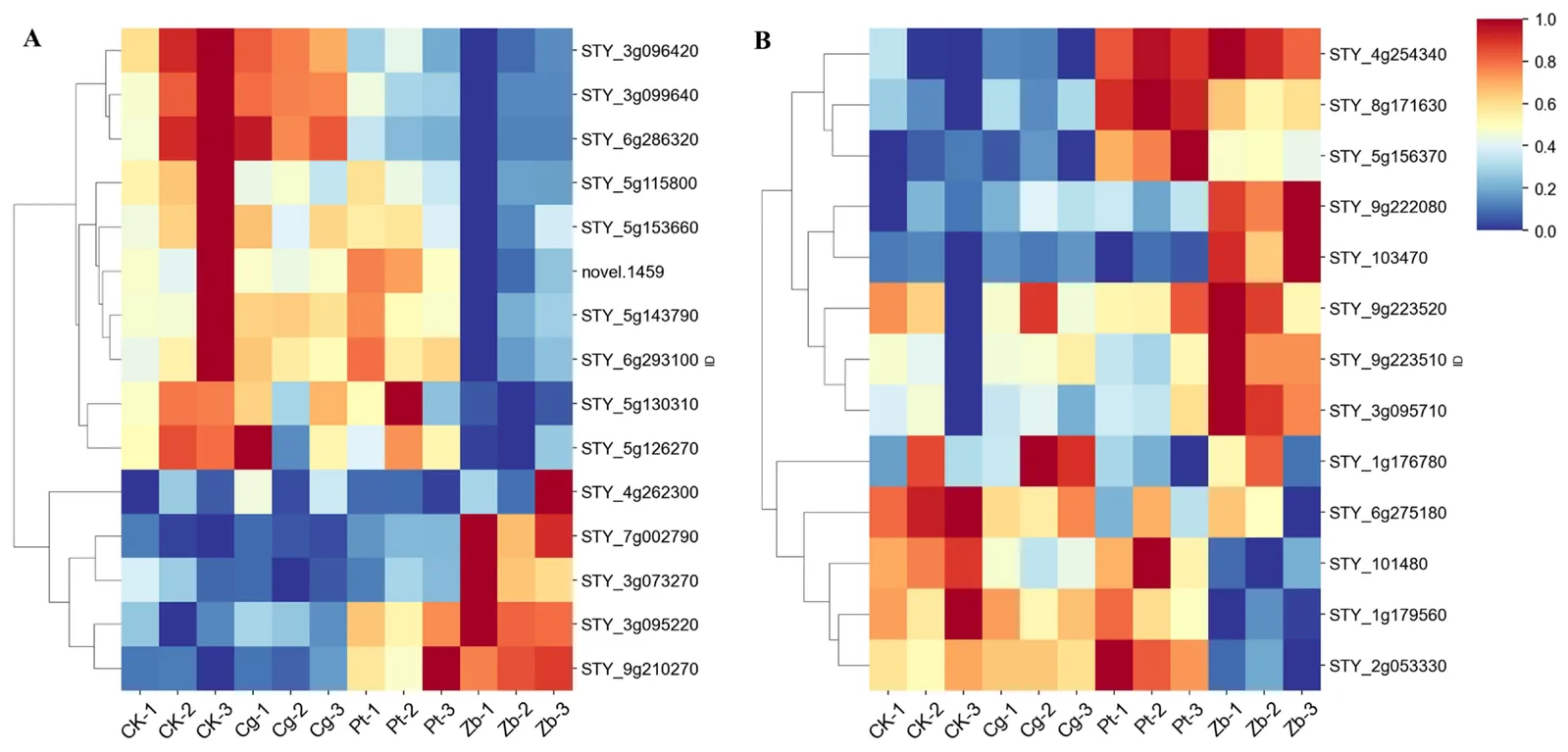
Expression profiles of candidate differentially expressed genes associated with carbohydrate transport and carbon metabolism (**A**), and ascorbate biosynthesis (**B**) in Shatian pomelo fruits across different grafting treatments. The color gradient from blue to red represents the normalized expression levels ranging from 0.0 to 1.0. Treatments are defined as follows: CK, self-rooted Shatian pomelo; Cg, Sour pomelo rootstock; Zb, Zhenban pomelo rootstock; Pt, Trifoliate orange rootstock.

Since carbohydrates serve as the essential carbon skeletons for ascorbate biosynthesis, we first profiled the alterations in sugar mobilization and energy utilization associated with the different rootstock treatments (Figure 5A and Supplementary Table S1). Notably, compared to the self-rooted CK control, a suite of sugar transporters exhibited significant upregulation specifically in the Zb rootstock. Specifically, the bidirectional sugar transporters SWEET15 (STY_7g002790) and SWEET10 (STY_7g016950) were highly induced in the Zb group relative to all other treatments. Genes encoding sugar transporter ERD6-like 16 (STY_9g210270) and ERD6-like 6 (STY_1g178420) also showed enhanced expression in the Zb treatment compared to the CK, suggesting a potential role in facilitating the transmembrane mobilization and intracellular redistribution of carbohydrates. Concomitantly, genes associated with starch metabolism and hexose utilization showed distinct transcriptional patterns across the treatments. Key enzymes involved in glycolysis and structural carbon metabolism experienced widespread transcriptional repression in the Zb rootstock compared to the high expression levels observed in the CK and Cg groups. Genes encoding cytosolic Fructose-bisphosphate aldolase 6 (STY_6g286320), Glucose-6-phosphate isomerase (STY_3g096420), and Pyrophosphate--fructose 6-phosphate 1-phosphotransferase (STY_3g099640) exhibited marked downregulation in the Zb group. Additionally, enzymes associated with cell wall polysaccharide synthesis, such as UDP-glucose 6-dehydrogenase 1 (STY_6g293100), were also repressed under the Zb treatment. These coordinated transcriptional shifts indicate a potential prioritization of sugar transport over local structural carbon consumption in the Zb rootstock, which may help provide the necessary carbon substrates for downstream secondary metabolic pathways.

Supported by this abundant carbon supply, the ascorbate metabolic network underwent a concerted transcriptional activation to maximize vitamin C accumulation (Figure 5B and Supplementary Table S1). Regarding the upstream biosynthesis pathway, the transcript abundances of multiple critical GDP-L-galactose phosphorylase (GGAP) genes, specifically STY_4g254340, STY_9g223510, and STY_9g223520, were markedly upregulated specifically in the Zb rootstock compared to the self-rooted control group. Concurrently, the downstream synthesis node governed by the L-galactono-1,4-lactone dehydrogenase (GLDH) gene STY_9g222080 also exhibited a significant expression increase in the Zb-treated fruits. Beyond direct biosynthesis, the capacity for intracellular transport and ascorbate recycling was similarly enhanced by the Zb rootstock. A suite of nucleobase-ascorbate transporter (NAT) genes (e.g., STY_103470, STY_3g095710, STY_1g182510) displayed significant transcriptional upregulation in the Zb group, facilitating the trans-membrane compartmentalization of ascorbate. Furthermore, the elevated expression of the monodehydroascorbate reductase (MDAR) gene STY_2g061220 specifically in the Zb rootstock actively promoted the regeneration of oxidized ascorbate back into its functional form. Conversely, key degradation enzymes exhibited a strongly repressed transcription pattern specifically under the Zb treatment. The primary ascorbate peroxidase (APX) genes (STY_8g171630, STY_1g176780, STY_6g275180) responsible for ascorbate consumption did not display any massive expression surges in the Zb group, while multiple ascorbate oxidase (ASOL) genes (STY_101480, STY_1g179560, STY_2g053330) were drastically downregulated relative to the CK, effectively blocking the primary route of ascorbic acid degradation. Ultimately, this robust activation of biosynthesis and recycling, paired with the strict restriction of oxidative degradation orchestrated by the Zb genotype, acts synergistically with the redirected carbon flux to drive the exceptional ascorbic acid surge observed in the Zb-grafted fruits.

### 3.6. Integrative Correlation Network Reveals Multi-Omics Synchronization Determining Fruit Quality

**Figure 6:**
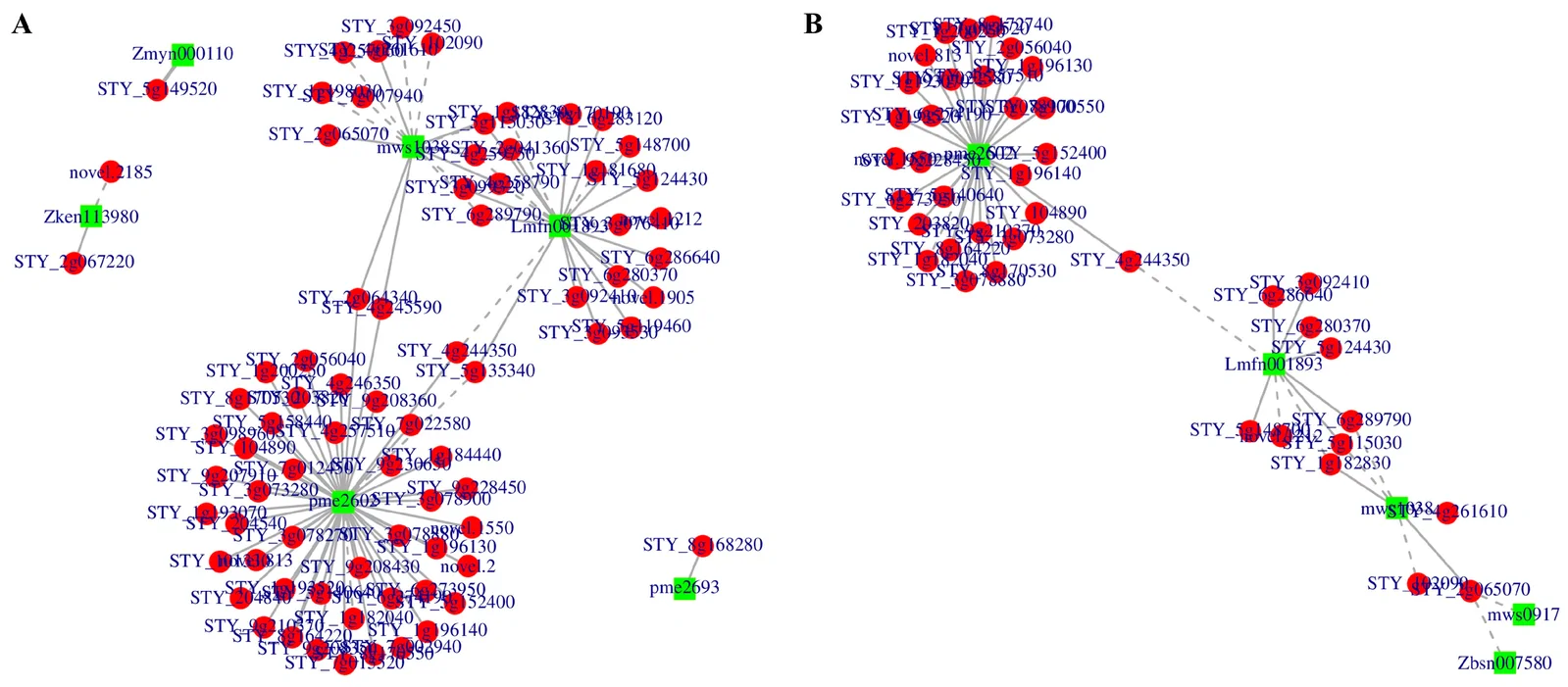
Integrative multi-omics correlation networks between differentially expressed genes (DEGs) and differentially accumulated metabolites (DAMs) in the Zb vs. CK comparison. (**A**) The correlation network enriched in primary “Metabolic pathways” (ko01100). (**B**) The correlation network enriched in “Biosynthesis of secondary metabolites” (ko01110). Red circular nodes represent genes, while green square nodes represent metabolites. Solid and dashed lines indicate positive and negative Pearson correlations between genes and metabolites, respectively (Pearson |r| > 0.8, *p* < 0.05).

Given that the preceding physiological and metabolomic analyses identified the Zb rootstock as the most effective treatment for enhancing soluble sugars and ascorbate while maintaining an optimal flavor balance, the subsequent integrative multi-omics network was exclusively focused on the targeted comparison between the Zb-grafted fruits and the CK. To holistically construct the regulatory architecture and identify the hub factors orchestrating Shatian pomelo fruit quality, a pairwise Pearson correlation analysis was performed between the highly responsive DEGs and DAMs within the Zb vs. CK group (Figure 6). Considering the prominent metabolomic shifts previously observed, this integrative analysis specifically targeted the core networks of “Metabolic pathways” (ko01100) and “Biosynthesis of secondary metabolites” (ko01110).

The integrated correlation networks unveiled a highly synchronized multi-omics landscape. Within the primary metabolic network (ko01100, Figure 6A), a robust co-expression cluster was identified linking key metabolic genes to carbohydrate intermediates. Notably, specific responsive DEGs exhibited remarkably high correlations with crucial sugar derivatives. For instance, the gene STY_2g067220 showed a strong positive correlation (r = 0.887, *p* < 0.001, Supplementary Table S2) with beta-D-Fructose 2-phosphate (Zken113980), a pivotal intermediate in sugar metabolism. Conversely, the transcript novel.2185 demonstrated a stringent negative correlation (r = −0.891, *p* < 0.001) with the same fructose derivative. Furthermore, the network highlighted intense regulatory hubs surrounding amino acid derivatives, such as the profound positive synchronization between STY_8g164220 and O-Phospho-L-serine (pme2602) (r = 0.954, *p* < 0.001). This tight mathematical synchronization provides compelling evidence that the local rootstock physically bridges the genetic transcriptional output with the enzymatic flux of primary carbohydrates, driving the macroscopic “high sugar” sink strength.

Even more compelling is the regulatory dynamic uncovered within the secondary metabolic network (ko01110, Figure 6B). The targeted suppression of flavonoids, initially observed in the global metabolome, was deeply substantiated at the correlation level. A distinct cluster of specific genes demonstrated profound negative correlations with key flavonoid intermediates. For example, the transcript STY_1g182830 exhibited a strict negative correlation (r = −0.839, *p* < 0.001) with leucocyanidin (Lmfn001893), a primary building block for proanthocyanidins. Similarly, STY_2g065070 showed a strong negative association (r = −0.812, *p* < 0.01) with the methylated flavonol syringetin (Zbsn007580). Other transcripts, such as STY_4g244350 and novel.1212, further reinforced this repressor network by negatively correlating with leucocyanidin (Lmfn001893) biosynthesis. This pervasive negative correlation network decisively indicates the presence of an active, rootstock-induced repressor mechanism. By transcriptionally inhibiting the metabolic flow towards these energy-intensive flavonoids, the Zb rootstock systematically ensures the strategic redirection of carbon skeletons.

## 4. Discussion

### 4.1. Rootstock Selection Drives Physiological Optimization of Fruit Quality

The application of suitable rootstocks is a fundamental cultivation strategy to overcome environmental constraints and improve the commercial value of fruit crops. Previous studies have extensively documented that citrus rootstocks can significantly influence the source-sink dynamics, water relations, and primary metabolite accumulation of the scion [19,20,21]. In commercial citrus production, conventional rootstocks often present a physiological dilemma regarding internal fruit quality. For instance, while trifoliate orange (*Poncirus trifoliata*) typically enhances soluble solids in sweet orange scions, it can also lead to undesirably high titratable acidity under certain pedoclimatic conditions, thereby reducing the overall maturity index and altering the flavor profile [22,23]. This phenomenon is often attributed to a mild rootstock-induced water deficit that concentrates all solutes, rather than a genuine optimization of carbon partitioning.

In the present study, we evaluated the physiological impacts of conventional rootstocks, trifoliate orange (Pt) and typical sour pomelo (Cg, *Citrus grandis*), alongside a local mutant, Zhenban pomelo (Zb). The introduction of the Zb rootstock maintained the fundamental morphological traits of Shatian pomelo while fundamentally optimizing the dry matter partitioning patterns, a phenomenon closely associated with rootstock-induced enhancements in scion sink strength [24]. Although total soluble solids (TSS) remained stable across all treatments, a comprehensive assessment incorporating titratable acidity (TA) and the TSS/TA ratio revealed profound differences in taste quality. The conventional Pt and Cg rootstocks caused a severe decline in the TSS/TA ratio, indicating significant flavor degradation. Because TSS was stable, this decline was driven mainly by TA, with Pt and Cg showing a less favorable TA profile than Zb and the self-rooted control. By contrast, Zb grafting exhibited a distinct TSS/TA trajectory: instead of declining, the ratio was maintained at an optimal level comparable to the self-rooted control. This difference is consistent with the biochemical profiles, which showed that Zb simultaneously elevated specific individual soluble sugars and ascorbic acid while significantly diminishing citric acid, thereby reducing TA and preserving the sugar-to-acid balance. These observations are highly consistent with previous studies indicating that rootstock genotypes profoundly modulate the ultimate sugar-to-acid balance and organic acid degradation in citrus scions [19,25]. This physiological achievement highlights the exceptional affinity and efficient source-sink communication between the Zhenban pomelo rootstock and the Shatian pomelo scion. Comparing our physiological outcomes with similar studies investigating citrus scion-rootstock interactions [26,27], it is evident that the Zb rootstock does not merely induce a passive stress-response, but rather actively enhances the fruit’s sink strength, ensuring an abundant and directed carbon supply for quality formation during the late developmental stages.

### 4.2. Targeted Metabolic Reprogramming Optimizes Carbon Allocation and Specific Secondary Pathways

The integration of transcriptomic and metabolomic datasets revealed that the introduction of the Zb rootstock induced a systemic yet highly precise metabolic reprogramming in Shatian pomelo fruits. While global multivariate analyses confirmed distinct spatial segregation of all grafted groups from the self-rooted control, it is particularly noteworthy that the Zb rootstock uniquely exhibited the lowest number of rootstock-specific differentially accumulated metabolites. Many previous metabolomic profiles of citrus scion-rootstock combinations typically report sweeping, non-specific global metabolic disruptions, where hundreds of metabolites fluctuate simultaneously as a generalized graft-induced stress response [26,27]. This targeted regulatory signature contrasts sharply with the broader and potentially chaotic physiological fluctuations typically induced by conventional rootstocks like Pt and Cg. According to the optimal partitioning theory [28], such precise metabolic modulation indicates that the Zb rootstock efficiently minimizes unnecessary biochemical redundancy. Instead of eliciting a generalized stress response, it directs cellular energy and resources toward highly specific physiological goals, thereby optimizing the overall metabolic efficiency of the fruit.

At the core of this targeted modulation is the profound reallocation of primary carbon fluxes. Our biochemical profiling demonstrated a massive enrichment of free sugars and their phosphorylated intermediates, particularly hexose phosphates, alongside a concurrent depletion of core tricarboxylic acid (TCA) cycle intermediates such as citrate and cis-aconitate. This inverse accumulation pattern between sugar derivatives and organic acids strongly suggests a rootstock-driven attenuation of respiratory carbon consumption. Previous metabolic flux models in citrus have proposed that restricted TCA cycle activity can prevent the excessive breakdown of imported photoassimilates [29]. By suppressing the carbon drain towards acidic intermediates, the Zb rootstock effectively preserves the structural carbon skeletons required for sweetening, channeling them directly into the biosynthesis of soluble sugars and their derivatives.

Beyond primary carbohydrate partitioning, the Zb rootstock dictates a masterfully coordinated trade-off within the secondary metabolic network. While existing metabolomic investigations in citrus often describe a parallel and simultaneous accumulation of flavonoids alongside other antioxidants under rootstock induction [30,31], our data reveal a highly distinct resource reallocation strategy. As observed in the class-specific metabolic reprogramming, the striking downregulation of energy-intensive flavonoids seamlessly explains the source of the redirected carbon flux, which is ultimately utilized to fuel the robust accumulation of ascorbic acid. Interestingly, while restricting broad flavonoid synthesis to conserve energy, the Zb rootstock specifically activated unique secondary defense and signaling pathways. This was notably characterized by the exclusive upregulation of steroids and tannins, as well as the universal accumulation of nucleotides. Steroids and tannins are crucial components for maintaining structural integrity and biotic stress resistance [32], while elevated nucleotide pools are closely associated with enhanced cellular signaling and umami flavor intensity in fruits [33]. This highly selective activation confirms that the Zb rootstock restricts generalized secondary metabolism to save carbon resources, but strategically invests in specific functional and health-promoting compounds. Ultimately, this tailored metabolic rewiring establishes a superior internal quality profile that balances enhanced sweetness and antioxidant capacity with targeted stress resilience.

### 4.3. Transcriptional Regulation Coordinates Carbohydrate Mobilization and Ascorbate Accumulation

The unparalleled metabolic optimization observed in Zb grafted fruits is driven by a highly orchestrated transcriptional reprogramming. As evidenced by the Kyoto Encyclopedia of Genes and Genomes enrichment analysis, the Zb rootstock specifically mobilized starch and sucrose metabolism networks. At the core of this sugar accumulation strategy is the robust activation of transmembrane carbohydrate transport. The profound upregulation of bidirectional sugar transporters, specifically SWEET and ERD6-like family members, provides direct molecular evidence for an accelerated phloem unloading and intracellular sugar redistribution. SWEET proteins have been extensively characterized as critical gatekeepers for sugar accumulation in fleshy fruits [34]. Concurrently, this enhanced sugar influx is protected from excessive respiratory consumption. The widespread transcriptional repression of key glycolytic enzymes, including fructose bisphosphate aldolase and glucose 6 phosphate isomerase, effectively restricts the structural and respiratory carbon drain. This dual mechanism of maximizing import while restricting catabolism perfectly explains the massive accumulation of mono and disaccharides at the physiological level.

The carbon skeletons conserved from the suppressed glycolytic and flavonoid pathways are efficiently funneled into the ascorbate biosynthesis network. Transcriptomic evidence confirms the hyperactivation of the L galactose pathway, primarily through the upregulation of multiple GDP L galactose phosphorylase (GGAP) genes. GGAP is universally recognized as the first committed rate-limiting step in plant ascorbate biosynthesis [35,36]. The concurrent upregulation of the terminal enzyme L galactono 1,4 lactone dehydrogenase (GLDH) further solidifies this directed metabolic flow. Beyond biosynthesis, the Zb rootstock actively enhances the subcellular compartmentalization of this critical antioxidant. The significant induction of nucleobase ascorbate transporter (NAT) genes facilitates the efficient transmembrane allocation of ascorbate, ensuring that the synthesized vitamin C is properly sequestered and utilized within specific organelles [37].

Crucially, the exceptionally high ascorbate phenotype in Zb grafted fruits is governed not only by de novo synthesis but also by a strict post biosynthetic regulatory mechanism. Contrary to previous assumptions that massive ascorbate accumulation solely relies on enhanced peroxidase activity, our transcriptome data revealed that key ascorbate peroxidase (APX) genes did not exhibit massive expression surges. Instead, the cellular redox homeostasis is maintained through the robust upregulation of monodehydroascorbate reductase (MDAR), an enzyme essential for recycling oxidized ascorbate back into its functional form [38]. Most importantly, the primary route of ascorbic acid degradation is aggressively blocked via the drastic downregulation of ascorbate oxidase (ASOL) genes. Ascorbate oxidase is the primary enzyme responsible for the irreversible breakdown of the ascorbate pool in the apoplast, and its suppression is a well-documented strategy for extending the shelf life and nutritional value of horticultural crops [39]. Ultimately, this synergistic network combining boosted biosynthesis, enhanced recycling, and blocked degradation acts as the molecular engine driving the targeted enhancement of ascorbic acid and soluble sugars observed in the Zb grafted fruits.

### 4.4. Integrative Multiomics Synchronization Determines Sink Strength and Resource Reallocation

As established by our physiological evaluations, the Zb rootstock specifically improved internal fruit quality by maintaining an optimal TSS/TA ratio through the reduction in titratable acidity, alongside the targeted accumulation of soluble sugars and ascorbic acid. The integration of transcriptomic and metabolomic data provides a comprehensive system level perspective on the potential molecular basis of these specific biochemical traits. Rather than reiterating the specific correlative pairs detailed previously, it is crucial to emphasize that the tight positive synchronization between metabolic gene expression and intermediate sugar accumulation in the Zb group is consistent with a highly active sink. In horticultural crops, intermediate sugar phosphates serve not merely as passive storage components but as potential signaling molecules that may influence sink strength and facilitate active phloem unloading [40,41]. The robust multiomics correlation networks observed in our study suggest that the Zb rootstock potentially supports these carbohydrate signaling cascades. Such coordinated metabolic gene expression appears to be a fundamental component associated with the elevated sugar levels observed, similar to findings in various fruit development models where enhanced transcriptomic synchronization correlates with improved sink capacity [30,42].

Furthermore, the integrative analysis of secondary metabolism provides insights into the potential molecular mechanism of resource reallocation. The pervasive negative correlation network observed between specific regulatory transcripts and flavonoid biosynthesis pathways suggests a targeted repressor mechanism. By transcriptionally downregulating the metabolic pathways leading toward these carbon-expensive secondary pools, the rootstock may help conserve structural carbon skeletons. This regulatory strategy aligns perfectly with advanced models of metabolic flux redirection in developing fruit, where limiting competitive secondary pathways can support maximal primary metabolite and essential antioxidant accumulation [43]. Similar multiomics repressor networks have been identified in other plant systems, illustrating that limiting excessive flavonoid production may be an efficient strategy to maintain cellular energy homeostasis under altered physiological conditions [44,45]. Ultimately, this precise genetic coordination potentially bridges the transcriptional output with enzymatic flux. It appears to optimize the internal fruit quality by shifting the metabolic priority away from broad secondary defense and toward the targeted carbohydrate accumulation of carbohydrates and extreme ascorbic acid synthesis, further supporting the immense commercial potential of the Zb rootstock.

## 5. Conclusions

In this study, we comprehensively evaluated the physiological and molecular impacts of three diverse grafting rootstocks (Pt, Cg, and Zb) on Shatian pomelo fruit quality. While conventional Pt and Cg rootstocks triggered broad nonspecific metabolic fluctuations, they severely degraded the flavor balance by lowering the TSS/TA ratio. In stark contrast, the Zb rootstock successfully maintained an optimal TSS/TA ratio comparable to the self-rooted control. Furthermore, it successfully decoupled specific carbohydrate accumulation from excessive acidity to yield fruits with enhanced levels of individual soluble sugars, diminished titratable and citric acid, and remarkably high ascorbic acid content. Integrative multiomics analyses suggested that this exceptional biochemical performance is associated with a targeted metabolic modulation rather than a chaotic stress response. By transcriptionally downregulating competitive pathways, specifically glycolysis and broad flavonoid biosynthesis, the Zb rootstock may help conserve vital structural carbon skeletons. This coordinated transcriptional shift, synchronized with the upregulation of SWEET sugar transporters and key enzymes in the L-galactose pathway, closely aligns with the targeted enhancement of carbohydrate mobilization and vitamin C production. Ultimately, the Zb rootstock appears to optimize internal fruit quality by shifting the metabolic priority away from broad secondary defense and toward targeted nutritional accumulation, establishing it as an elite germplasm resource and a theoretical model for source sink dynamics in grafted fruit crops. Future investigations will focus on the functional validation of key regulatory genes associated with this metabolic shift and the evaluation of the long-term agronomic stability of the Zb rootstock across diverse agroclimatic regions to fully translate these theoretical insights into commercial citrus breeding.

## Figures and Tables

**Table 1:** Effects of distinct grafting rootstocks on the basic morphological and quality traits of Shatian pomelo fruits.

Treatment	Single Fruit Weight (kg)	Total Soluble Solids (TSS, Brix)	Dry Matter Content (%)	TSS/TA
Pt ^1^	1.06 ^a2^	12.47 ^a^	27.7 ^c^	9.32 ^c^
Cg	1.09 ^a^	12.53 ^a^	28.9 ^c^	9.90 ^b^
Zb	1.10 ^a^	12.51 ^a^	30.9 ^b^	10.31 ^a^
CK	1.08 ^a^	12.44 ^a^	34.6 ^a^	10.54 ^a^

^1^ Treatments are defined as follows: CK, self-rooted Shatian pomelo; Cg, Sour pomelo rootstock; Zb, Zhenban pomelo rootstock; Pt, Trifoliate orange rootstock. ^2^ Different letters indicate significant differences at *p* < 0.05 according to Duncan multiple range test.

## Data Availability

The sequence data have been submitted to the NCBI Sequence Read Archive (https://www.ncbi.nlm.nih.gov/sra, accessed on 12 August 2026) under BioProject accession number PRJNA1511532. Formal SRA accession numbers will be supplemented after data processing is completed.

## Supplementary Materials

The following supporting information can be downloaded at https://www.mdpi.com/article/10.3390/metabo16090697/s1, Table S1: Transcriptomic expression data and functional annotations of candidate genes associated with carbohydrate metabolism and ascorbate biosynthesis; Table S2: Multiomics correlation analysis between differentially expressed genes and key metabolites mapped to primary (ko01100) and secondary (ko01110) metabolic pathways.

## Abbreviations

The following abbreviations are used in this manuscript:

Pt	Trifoliate orange
Cg	Sour pomelo
Zb	Zhenban pomelo
CK	Self-rooted Shatian pomelo
SWEET	Sugars Will Eventually be Exported Transporters
SUTs	Sucrose transporters
TCA	Tricarboxylic acid
CS	Citrate synthase
ACL	ATP citrate lyase
GGAP (VTC2)	GDP L galactose phosphorylase
GLDH	L galactono 1,4 lactone dehydrogenase
NAT	Nucleobase ascorbate transporter
MDAR	Monodehydroascorbate reductase
ASOL	Ascorbate oxidase
APX	Ascorbate peroxidase
KEGG	Kyoto Encyclopedia of Genes and Genomes
GO	Gene Ontology
FPKM	Fragments per kilobase of transcript per million mapped reads
ATP	Adenosine triphosphate
UDP	Uridine diphosphate
